# Long non-coding RNAs associated with non-small cell lung cancer

**DOI:** 10.18632/oncotarget.20088

**Published:** 2017-08-09

**Authors:** Yuting Zhan, Hongjing Zang, Juan Feng, Junmi Lu, Lingjiao Chen, Songqing Fan

**Affiliations:** ^1^ Department of Pathology, The Second Xiangya Hospital, Central South University, Changsha, Hunan, China

**Keywords:** NSCLC, lncRNAs, chemotherapy, radiotherapy, molecular therapy

## Abstract

Lung cancer, with 80–85% being non-small cell lung cancer (NSCLC), is the leading cause of cancer-related death in both men and women. Long non-coding RNAs (lncRNAs), always defined as non-protein-coding RNA molecules longer than 200 nucleotides, are now thought as a new frontier in the study of human malignant diseases including NSCLC. As researches continue, increasing number of roles that lncRNAs play in NSCLC has been found, and more and more evidences show lncRNAs have a close relationship with patients’ response to radiochemotherapy or molecular therapy. The aim of this review is to disclose the roles that lncRNAs play in NSCLC and how lncRANs influence the treatment of NSCLC.

## INTRODUCTION

Lung and bronchus cancer leads to most cancer-related death, with an estimation about 85,920 and 72,160 death in men and women respectively in America, and it was also reported that there were totally 224,390 American people being diagnosed with lung and bronchus cancer in 2016 [1]. Looking around the world, greater than one-third of all newly diagnosed lung cancers occurred in China, resulting in a large social and economic burden. According to the annual report on the status of cancer in China, in total, 651,053 patients were newly diagnosed with lung cancer in 2011, including 441,364 men and 209,689 women [2]. Lung and bronchus cancer is usually classified into NSCLC and small cell lung cancer (SCLC) accounting for approximately 80% and 20% respectively, the former of which is traditionally treated with surgery combined with or without radiochemotherapy [3].

Long non-coding RNAs (lncRNAs), always defined as non-protein-coding RNA molecules longer than 200 nucleotides, are not thought as transcriptional “noise” any more, and have been regarded as a new frontier in the study of human malignant diseases such as brain, breast, prostate, liver, ovary, esophagus tumors and other kinds of diseases like Fragile X syndrome, Alzheimer's disease and *etc* [4, 5]. LncRNAs, on the one hand, can regulate the expression of nearby protein-coding genes, and on the other hand, they themselves can serve key regulatory roles. In Jeremy's review, there are basically eight models for lncRNAs to influence the gene expressions, and eventually play the biological roles [6]. As researches continue, it has been increasingly recognized that its dysregulations contribute to the development and progression of lung and bronchus cancer [7]. We conduct a comprehensive review of the published literatures focusing on the roles that lncRNAs play in the presence and development of NSCLC, retrospect the relationship between lncRNAs and radiochemotherapy as well as molecular targeted therapy of NSCLC, and discuss future directions about lncRNAs in the researches of NSCLC.

### Roles of LncRNAs in NSCLC

It has been proved that the abnormal expression of lncRNAs has a close relationship to NSCLC. Here is a quick review of some popular and well-studied lncRNAs related to NSCLC.

### *MALAT1* and NSCLC

The *metastasis-associated lung adenocarcinoma transcript 1* (MALAT1), also known as *nuclear-enriched abundant transcript 2 (NEAT2)*, was firstly identified in 2003 by subtractive hybridization as prognostic parameters for patient survival in stage I of NSCLC. *MALAT1*, more than 8000 nt expressed from chromosome 11q13, was detected not only in NSCLC, but also in some normal tissues such as pancreas, lung, prostate, ovary, colon, placenta, spleen, small intestine, kidney, heart, liver, testis and brain [8]. It was believed that *MALAT1* expression levels were associated with patient survival by affecting genes involved in cancer like cellular growth, movement, proliferation, signaling, and immunoregulation [9]. Q-PCR was performed to confirm that the *MALAT1* were upregulated in cancerous tissues than that in adjacent normal tissues [10]. And *in vitro* studies, migration and clonogenic growth could be suppressed by RNA-interference-mediated suppression of *MALAT1* in A549 cells, while forced expression of *MALAT1* in NIH 3T3 cells significantly increased migration [9]. What's more, the level of *MALAT1* was higher in brain metastasis and it was increased in highly invasive subline of brain metastasis lung cancer cells, which was speculated on account for epithelial-mesenchymal transition (EMT) [11]. For the mechanism, there is no final conclusion until now. Some scholars thought it was regulated by DNA methylation [12] and some suspected *MALAT1* of contributing to NSCLC by upregulating the expression of Bcl-2 and its interacting proteins [13]. Besides, it is reported that *MALAT1* regulates alternative splicing (AS) of pre-mRNAs, which is a key step in the regulation and diversification of gene function, by controlling the levels of active serine/arginine (SR) proteins and the distributions to nuclear speckles [14]. Above all were recognized as modes of action for *MALAT1*: regulation of gene expression or alternative splicing [15].

### *HOTAIR* and NSCLC

*HOX antisense intergenic RNA (HOTAIR)*, a 2.2 kilobase noncoding RNA residing in the *HOXC locus*, was firstly identified in 2007. Rinn *et al*. proved in that paper that it might regulate gene expression in HOX loci in *cis* or *trans*; alternatively, it might be the act of antisense transcription in the *HOXC locus* [16]. And it was widely accepted that *HOTAIR* regulated gene expression by EZH2 (a subunit of PRC2), which led to histone H3 lysine 27 trimethylation of the *HOXD locus*, and it also could mediate chromosomal remodeling [17, 18]. In addition, it was confirmed that *HOTAIR* was highly expressed in both NSCLC samples and cell lines compared with adjacent tissues and it indicated a poor prognosis [19]. In the mechanism of how *HOTAIR* contributed to NSCLC, it was thought that *HOTAIR* might facilitate the tumor development but not the carcinogenesis of NSCLC [20]. In the meantime, some scholars found that *HOTAIR* modified the promoter of *p53* and enhanced histone H3 lysine 27 trimethylation, which showed a negative relationship between *HOTAIR* and *p53* in NSCLC cells [21]. What's more, it was reported that *HOTAIR* can activate Wnt/β-catenin signaling pathway in esophageal squamous cell carcinoma [22]. In addition, *HOTAIR* could involve in EMT, and also worked as competitive endogenous RNAs (ceRNAs) [23].

### *HOTTIP* and NSCLC

*HOXA distal transcript antisense RNA (HOTTIP)* is an antisense non-coding transcript located at the distal end of HOXA gene cluster. It was regarded as key intermediates to transmit information from higher order chromosomal looping into chromatin modifications, and thus coordinated long range gene activation, which was associated with the WDR5/MLL complex to drive the H3 lysine 4 trimethylation and gene transcription [24, 25]. And it was identified as the most significantly up-regulated lncRNAs in human primary hepatocellular carcinoma even in early stage [26]. In NSCLC, *HOTTIP* expression was higher than corresponding adjacent normal tissues and contributed to cell proliferation and migration, which was by regulating *HOXA13* and functioning as oncogene [27].

Besides these relatively common and popular lncRNAs mentioned above, there were still some other lncRNAs proved having close relationship to NSCLC which will be presented in Table 1 and Table 2.

**Table 1:** Overexpressed or upregulated lncRNAs in NSCLC tissues or cell lines and their functions and probable mechanism

LncRNA	Function in NSCLC	probable mechanism	cition
AGAP2-AS1	negatively correlated with poor prognostic outcomes	repressed tumor-suppressor LATS2 and KLF2 transcription	[28]
ATB	presented a lower survival probability		[29]
TCF7	promoted invasion and self-renewal	TCF7 upregulated EpCAM expression through functioning as a competitive endogenous RNA (ceRNA)	[30]
SBF2-AS1	increased the proliferation of NSCLC cells	negatively regulated P21	[31]
FOXD2-AS1	promoted NSCLC cell growth and NSCLC tumor progression	Wnt/β-catenin signaling	[32]
HOXA11-AS	promoted development and progression of NSCLC	regulated the expression of various pathways and genes, especially DOCK8 and TGF-beta pathway.	[33]
PCAT-1	played an oncogenic role in NSCLC progression		[34]
BCAR4	associated with poorer 5-year overall survival rate of NSCLC patients		[35]
CCAT2	promoted tumorigenesis	over-expression of Pokemon	[36]
00511	functioned as an oncogene	acted as a modular scaffold of EZH2/PRC2 complexes	[37]
XIST	associated with shorter survival and poorer prognosis	by epigenetically repressing KLF2 expression	[38]
NEAT1	correlated with poor prognosis	inhibition of miR-377-3p/ E2F3 axis.	[39]
ANRIL	correlated with advanced tumor–node–metastasis stage and greater tumor diameter		[40]
ZFAS1	an independent prognostic factor for poor survival of NSCLC patients		[41]
SNHG1	associated with a poor overall survival	inhibited miR-101-3p and activated of Wnt/β-catenin signaling pathway	[42]
RGMB-AS1	correlated with differentiation, TNM stage, and lymph node metastasis	by regulating RGMB expression though exon2 of RGMB	[43]

**Table 2:** Lower expressed or downregulated lncRNAs in NSCLC tissues or cell lines and their functions and probable mechanism

LncRNA	Function in NSCLC	probable mechanism	cition
TUSC7	associated with worse overall survival		[44]
CASC2	independent predictor for overall survival of NSCLC		[45]
GAS5	indicated a poor prognosis and regulated cell proliferation		[46]
TUG1	related to the proliferation of NSCLC cells	TUG1 RNA could bind to PRC2 in the promotor region of CELF1 and negatively regulated CELF1 expressions	[47]
AK126698	inhibited the proliferation and migration	inhibited the activation of Wnt/β-catenin pathway	[48]
GAS5-AS1	regulated NSCLC cell migration and invasion	through regulation of EMT	[49]

### LncRNAs associated with radiochemotherapy of NSCLC

### LncRNAs and chemotherapy

As is well-known, DNA damage repair (DDR) mechanisms, such as single-strand break, double-strand break, bulky adducts, base mismatches and base alkylation, are playing important roles to maintain genomic stability. Thanks to these precise modulations, cells could maintain genomic integrity confronted with numerous physical or chemical or even deadly strikes [50–52]. Platinum, a kind of chemical elements, could also cause DNA damage, especially in tumor cells, where the DDR is not complete.

Since found by Rosenbery in 1969, platinum was widely used in clinical practice including chemotherapeutics of NSCLC, which benefited a lot of incipient or advanced patients [53, 54]. Tumor cells, which proliferated more rapidly than normal ones, could be influenced directly by anticarcinogen, thus stopping equal division of DNA to next generation [55]. Binding of platinum and genomic DNAs in cell nucleus was thought to play important roles in cancer therapy, which influenced transcription and DNA replication and finally led to death of tumor cells [56]. However, the use of platinum was limited due to its toxicity, drug resistance, and some other side effects, which was demonstrated closely to lncRNAs [57, 58]. The polymorphisms of lncRNAs such as *HOTTIP, CCAT2, H19, HOTAIR, MALATI, ANRIL* and CASC8 were proved significantly associated with lung cancer risk or platinum-based chemotherapy response [58, 59]. It was reported that *HOTAIR* was significantly upregulated in cisplatin-resistant NSCLC cells both *in vitro* and *in vivo*, and it could enhance tumor cell proliferation, influence G0/G1 cell-cycle arrest, and decrease tumor cell apoptosis. Further studies showed that overexpression of *HOTAIR* could promote tumor sphere formation, which upregulated expression of the tumor stem cell-related biomarkers such as Nanog, Oct3/4, Sox2, c-Myc, β-catenin, and Klf4 [60, 61]. It was found inverse correlation between *HOTAIR* and *p21* [62], the latter of which was proved as a negative regulator of the cell cycle [63]. What's more, lncRNA *H19* had a negative relationship with cisplatin-based chemotherapy response, the enhancement of which associated with metastasis, induction of G0/G1 cell-cycle arrest, cell proliferation, and increased apoptosis [64]. Other lncRNAs were reported to relate to response to chemotherapy such as *HOTTIP* in osteosarcoma [65], *MALAT1* in laryngeal squamous cell carcinoma [66], and *ANRIL* in nasopharyngeal carcinoma [67].

### LncRNAs and radiotherapy

Radiotherapy is essential in most patients especially with advanced stage NSCLC, usually sequentially or simultaneously combined with surgery, chemotherapy and molecular therapy [68, 69]. Radioactive rays could cause a series of physical, chemical and biological damages to both tumor cells and normal cells, of which doctors make use, to cure cancer by reducing radiological dose of normal tissues and increasing that of tumor cells [70]. LncRNAs were proved to involve in the DNA damage response after radioactive rays, which might lead to the failure of radiotherapy [71]. However, the detailed mechanisms about lncRNA and resistance to radioterapy haven't been found, but it is certain that miRNAs do involve in the radioresistance of head and neck cancer [72]. And it is deserved to investigate whether there are some relationship between lncRNAs and radioresistance in NSCLC.

### LncRNAs associated with molecular targeted therapy of NSCLC

Basically speaking, there are three kinds of targeted therapies for NSCLC so far, namely EGFR tyrosine kinase inhibitors (EGFR-TKIs), antiangiogenic agents and Programmed cell death protein 1 inhibitors, which brings hopes and prospects to patients suffering NSCLC.

### EGFR and LncRNAs

Epidermal growth factor receptor (EGFR) super-family has been regarded as a therapeutic target to NSCLC. It was firstly reported in 2004 that the mutations of patients who were sensitive to gefitinib were around the ATP-binding pocket of the tyrosine kinase domain, which were small, in-frame deletions or amino acid substitutions [73]. Adenocarcinomas from never smokers were easier to acquire this kind of gene mutation, which meant they were more sensible to gefitinib or erlotinib [74]. According to statistics from Ohashi K, patients whose metastatic tumors identified EGFR mutations were expected to live longer than 2 years [75]. Following the use of these drugs, most patients who were initially sensitive to EGFR TKIs eventually acquired inevitable resistance after long-term therapy. And the EGFR T790M secondary mutation, which substituted methionine for threonine at residue 790, was firstly reported only a year later after the discovery of EGFR-TKIs [76]. The EGFR T790M secondary mutation was identified from patients who were not sensitive to EGFR-TKIs up to 68%. Besides, there were some other rare mutation such as D761Y, L747S, and T854A discovered in 2006, 2007, 2008 respectively [77–79]. In addition, mechanisms such as MET amplification, PTEN downregulation, CRKL amplification, High-level expression of HGF, FAS–NFƙB activation, EMT, and transformation to small cell lung cancer, were also working in the EGFR-TKIs resistance [80].

Few papers mentioned the relationship between lncRNAs and EGFR mutations in NSCLC. *Urothelial cancer-associated 1 (UCA1)*, a long non-coding RNA highly specific to Bladder transitional cell carcinoma (TCC), was proved to have a close relationship to colorectal, gastric, ovarian cancer and NSCLC [81, 82]. *UCA1* acted as an oncogenic role in NSCLC and it was proved that the expression of *UCA1* in NSCLC samples was significantly higher compared with adjacent tissues partly through competitively ‘sponging’ miR-506-3p [83]. *UCA1* was also highly expressed in patients with acquired resistance to EGFR-TKIs, and further studies showed that it was related to non-T790M by activating AKT/mTOR pathway and EMT [84].

LncRNA BC087858 could stimulate acquired resistance to EGFR-TKIs in NSCLC and might contribute to a shorter progression-free survival (PFS) [85]. Further study showed that lncRNA BC087858 could induce non-T790M mutation acquired resistance to EGFR-TKIs by activating PI3K/AKT and MEK/ERK pathways and EMT via upregulating ZEB1 and Snail and eventually promoted cell invasion [86].

**Figure 1:**
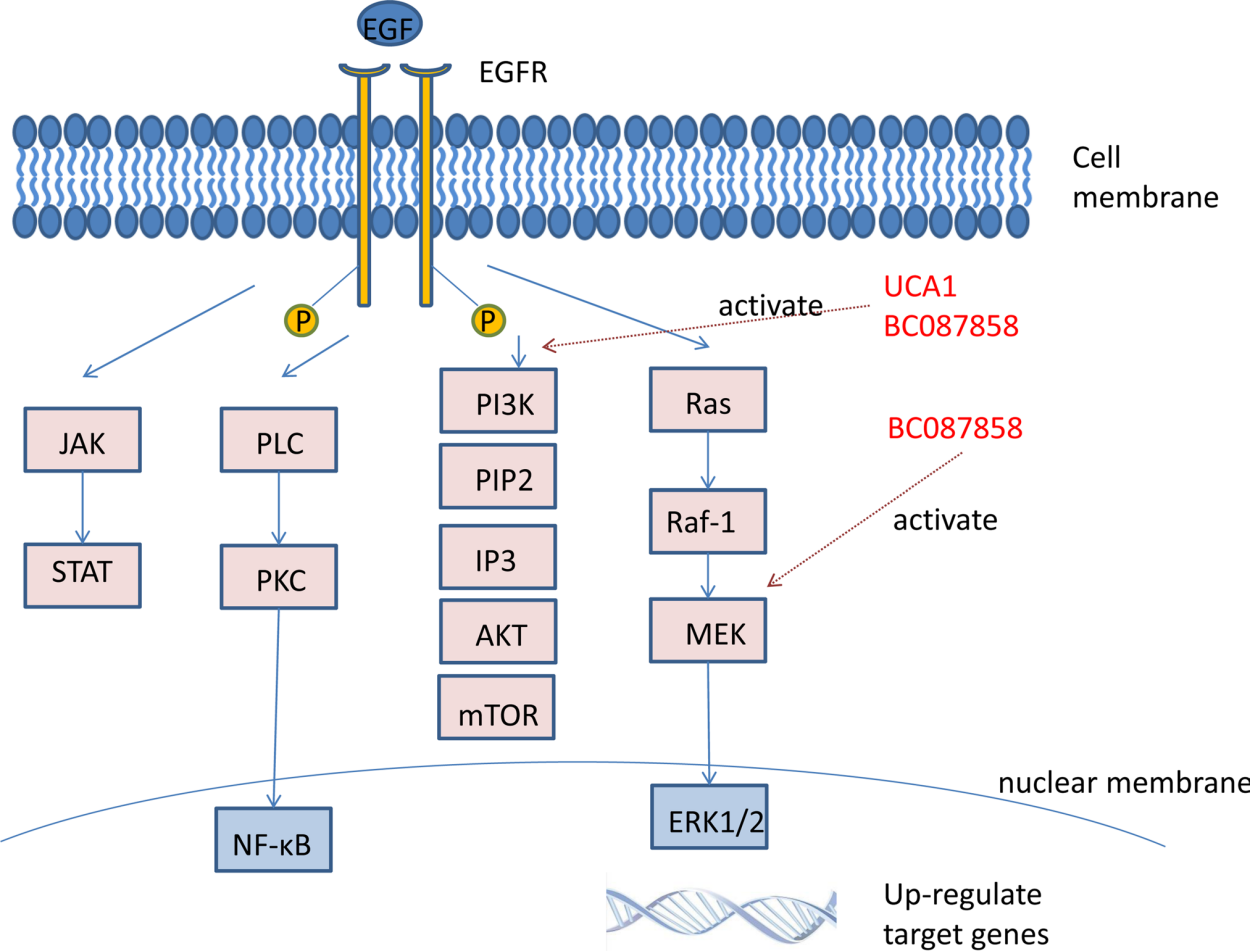
LncRNAs associated non-T790M mutation of NSCLC UCA1 activated AKT/mTOR pathway and related to non-T790M mutation. LncRNA BC087858 induced non-T790M mutation by activating PI3K/AKT and MEK/ERK pathways.

Not only single lncRNA could influence EGFR-TKIs resistance, but also lncRNAs could interact each other to affect the sensitivity to targeted therapy. The over and lower expression of *CASC9* and lncRNA 00277 were respectively negative to sensitivity to gefitinib in PC9G2 cells, and taken together, it was reported that they contributed to NSCLC cells EGFR-TKI resistance through interacting with their co-expressed gene, namely *PcGs*, and affected different biological pathways [87] (Figure 1).

### PD-1, PDL-1 and LncRNAs

The microenvironment of malignant cells were gaining highlight to the treatment of tumors and many labs were concentrating on finding ways to make immune cells kill cancer cells. T cells were the major cells to fight or kill malignant cells, and the activation of T cells was partly depending on immune checkpoints [88, 89]. Programmed cell death protein 1 (PD-1) and programmed cell death protein ligand 1 (PDL-1) are two of key components of immune checkpoints. It was widely accepted that the engagement of PD-1 by PDL-1 could suppress immune responses and consequently led to immune evasion [90, 91]. Therefore the study of PD-1 and PDL-1 is now offering new important opportunities for the therapy of cancer.

PDL-1 expression of tumor was significantly associated with a shorter PFS [92], and for the researches of its receptor, namely PD-1, showed that cumulative response rates to anti-PD-1 antibody were 18% among NSCLC patients according to a clinical trial in America (14 of 76 patients), which provided a kind of new method to NSCLC treatment [93]. Some scholars suggested the combination of EGFR-TKIs and immune checkpoints inhibitors, but due to the toxicity of this kind of combination, it aroused a lot of controversy [94, 95].

**Figure 2:**
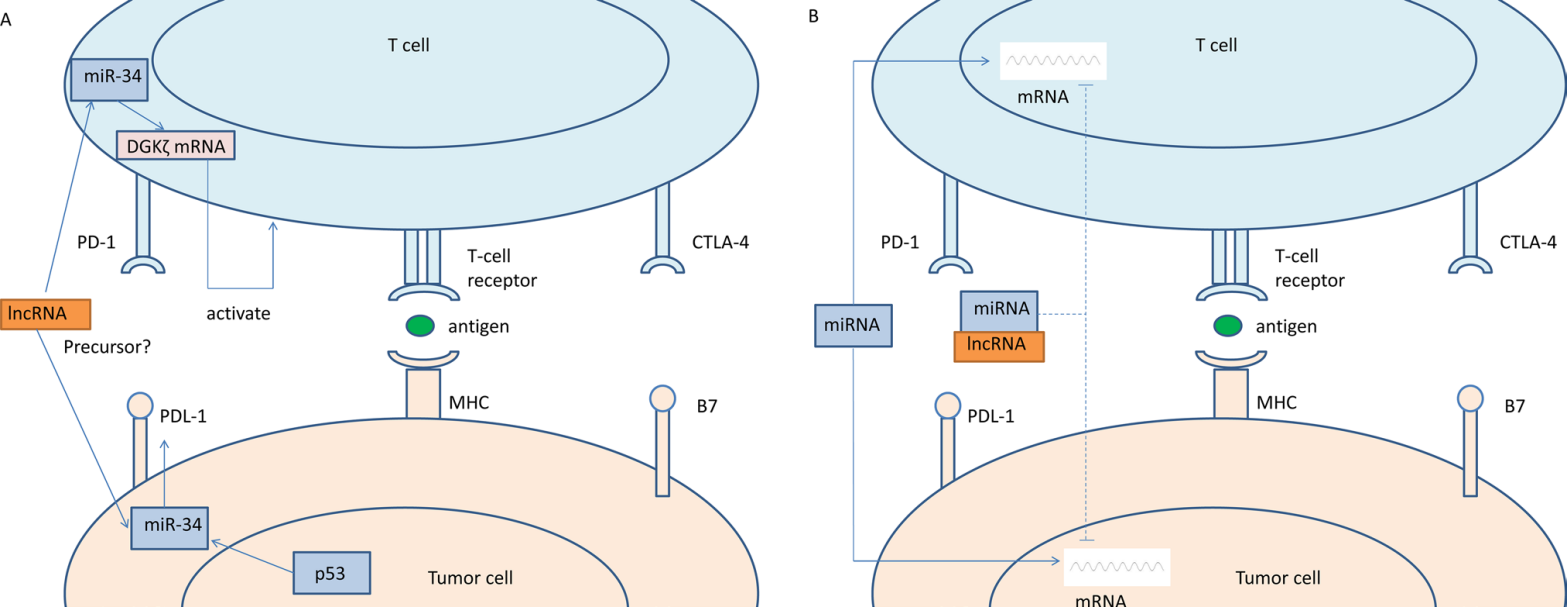
Hypothesis: LncRNAs associated the patients’ response to anti-PD-1 or anti-PDL-1 treatment (**A**) LncRNAs could be precursors of miRNAs. *P53* regulated PDL-1 via miR-34, and miR-34 enhanced T cell activation via targeting diacylglycerol kinase ζ. (**B**) LncRNAs could act as ceRNAs to alter the distribution of miRNA molecules on their targets.

Although many evidences showed that PD-1/PDL-1 had a promising future to treat with NSCLC, we knew little about the regulation about expression of PD-1/PDL-1. Previous studies demonstrated that PD-1.5 C/T significantly increased advanced NSCLC risk and potentially related to NSCLC susceptibility in Chinese Han population [96]. However, to our knowledge, there was no report about the relationship between PD-1/PDL-1 and lncRNAs in NSCLC, and only one paper was found to reveal that the co-expression of lncRNA *AFAP1-AS1* and PD-1 predicted poor prognosis of nasopharyngeal carcinoma (NPC) [97]. At present, it was found that *p53* regulated PDL-1 via miR-34, which directly binded to the PDL-1 3′untranslated region in models of NSCLC [98]. What's more, it was also reported that miR-34 enhanced T cell activation via targeting diacylglycerol kinase ζ [99] (Figure 2A). Because the expression of miRNAs was quite specific to distinct tumors, and they could affect early regulation of immune responses, they were regarded as suitable molecules for cancer therapy [100]. In addition, lncRNAs could be precursors of miRNAs and act as ceRNAs to alter the distribution of miRNA molecules on their targets [6, 101] (Figure 2B), for example, it was found that lncRNA *ARSR* acted as a ceRNA for miR-34 and miR-449 and finally promoted Sunitinib resistance in renal cancer [102]. Thereby we can speculate that lncRNAs probably influence the patients’ response to anti-PD-1 or anti-PDL-1 treatment.

### Antiangiogenic agents and LncRNAs

Researches showed that tumor growth and metastatic potential partly related to tumor angiogenesis. Vascular endothelial growth factor (VEGF), inducing angiogenesis *in vivo*, was expressed in most solid cancers including NSCLC [103]. Bevacizumab, a humanized monoclonal antibody, could block the binding of VEGF-A isoforms to VEGF receptors and therefore against tumors [104]. The Food and Drug Administration approved bevacizumab for the treatment in first-line metastatic setting of colorectal cancer, non-small cell lung cancer and breast cancer, and randomized controlled trials (RCTs) showed that bevacizumab-based regimens revealed significantly increased overall survival (OS) [105, 106].

For the relationship between VEGF and lncRNAs, it was proved that *MALAT1* could promote angiogenesis and immunosuppressive properties of mesenchymal stem cells by inducing VEFG in preeclampsia [107]. More direct evidence is that when lincRNA *p21* was inhibited, the expressions of angiogenesis-related genes were downregulated and lincRNA-p21-inhibited cells were observed to secrete less VEGFA than controls did [108].

## CONCLUSIONS AND FUTURE DIRECTIONS

With the deep research, lncRNAs are not regarded as transcriptional “noise” any more, and they are thought to be a new frontier for many diseases including malignant tumors. Based on existing evidences, lncRNAs are playing important roles in the presence and development of NSCLC, which leads to most cancer-related death. What's more, over or lower expression of lncRNAs could alter the ability of cellular growth, movement, proliferation, signaling, immunoregulation and invasion, consequently to influence the prognostication of cancer. Besides, it has a close relationship between lncRNAs and the response to radiochemotherapy or molecular targeted therapy, by which ulteriorly affect the prognostication of NSCLC. Following the development of body fluid detection, lncRNAs test will not only be applied into operative tissues, but also in blood, urine and other body fluid and will have a better predictive and diagnostic function [109].

In the future, further studies would be concentrated on the following aspects: (1) identifying new lncRNAs (2) discovering more functions of lncRNAs (3) detecting more relationships with miRNAs and other non-coding RNAs (4) seeking more probable pathways that lncRNAs influence the gene transcript or protein expression (5) looking for possibility of lncRNAs as therapeutic targets (6) developing more precise and reliable ways to detect lncRNAs in body floods.

### Ethics approval and consent to participate

Not applicable.

## Abbreviations

NSCLC: non-small cell lung cancer
lncRNA: long non-coding RNA
SCLC: small cell lung cancer
MALAT1: metastasis-associated lung adenocarcinoma transcript 1
NEAT2: nuclear-enriched abundant transcript 2
EMT: epithelial-mesenchymal transition
AS: alternative splicing
SR: serine/arginine
HOTAIR: HOX antisense intergenic RNA
ceRNA: competing endogenous RNA
HOTTIP: HOXA distal transcript antisense RNA
SBF2-AS1: SBF2 antisense RNA 1
HOXA11-AS: HOXA11 antisense RNA
PCAT-1: Prostate cancer-associated transcript 1
BCAR4: breast cancer anti-estrogen resistance 4
CCAT2: colon cancer-associated transcript 2
XIST: X inactivate-specific transcript
NEAT1: nuclear enriched abundant transcript 1
SNHG1: small nucleolar RNA host gene 1
TUSC7: Tumor suppressor candidate 7
CASC2: cancer susceptibility candidate 2
GAS5: growth arrest-specific transcript 5
UCA1: Urothelial cancer-associated 1
AK126698: lncRNA AK126698
DDR: DNA damage repair
CCAT2: Colon Cancer Associated Transcript 2
EGFR-TKIs: Epidermal growth factor receptor tyrosine kinase inhibitors
EGFR: Epidermal growth factor receptor
MET: Mesenchymal-epithelial transition
TCC: transitional cell carcinoma
PFS: progression-free survival
AFAP1-AS1: actin filament-associated protein 1 antisense RNA
NPC: nasopharyngeal carcinoma
VEGF: Vascular endothelial growth factor
RCT: randomized controlled trials
OS: overall survival
